# Acute Levothyroxine Overdose Followed by Serial Monitoring of Total Thyroxine and Thyroid‐Stimulating Hormone in a Hypothyroid Dog

**DOI:** 10.1002/vms3.71307

**Published:** 2026-11-12

**Authors:** Soomin Park, Jiyeon Seo, Taeho Lee, Woonchan Ahn, Woojin Song, Aryung Nam

**Affiliations:** ^1^ Department of Veterinary Internal Medicine College of Veterinary Medicine, Konkuk University Seoul South Korea; ^2^ Signature Animal Medical Center Seoul South Korea

**Keywords:** canine, hypothyroidism, levothyroxine overdose, thyroid‐stimulating hormone

## Abstract

Reports of acute levothyroxine overdose in dogs are rare with limited monitoring data. An 8‐year‐old spayed female Pomeranian receiving long‐term hypothyroidism treatment presented 9 h after ingesting a one‐month supply of levothyroxine (3 mg; 1.13 mg/kg). Except for transient tachycardia, the physical examination revealed unremarkable findings. The total thyroxine (T4) level exceeded the upper limit of quantification. Levothyroxine was discontinued, and activated charcoal and intravenous fluid were administered. The dog showed normal vital signs and no further clinical abnormalities during the 5‐day hospitalisation. The total T4 concentration decreased and normalised by 73 h after the overdose. The canine thyroid‐stimulating hormone (cTSH) concentrations were initially normal but exceeded the upper reference limit at 98 h. The patient was discharged on Day 5. Two days later, levothyroxine was resumed because the dog exhibited signs of recurrent hypothyroidism with persistently elevated cTSH levels. Hypothyroidism was well‐controlled for 6 months. To our knowledge, this is the first report to document a levothyroxine overdose in a hypothyroid dog using daily total T4 and cTSH monitoring. These findings suggest that acute overdose is less severe than chronic levothyroxine exposure.

## Graphical abstract

An 8‐year‐old hypothyroid dog presented following acute ingestion of a 1‐month supply of levothyroxine, with transient tachycardia. Serial monitoring of total T4 and cTSH was performed during the 5‐day hospitalisation; total T4, which initially exceeded the upper limit of quantification, normalised by 73 h after the overdose, followed by an increase in cTSH at 98 h. Hypothyroidism remained well controlled during 6 months of follow‐up.

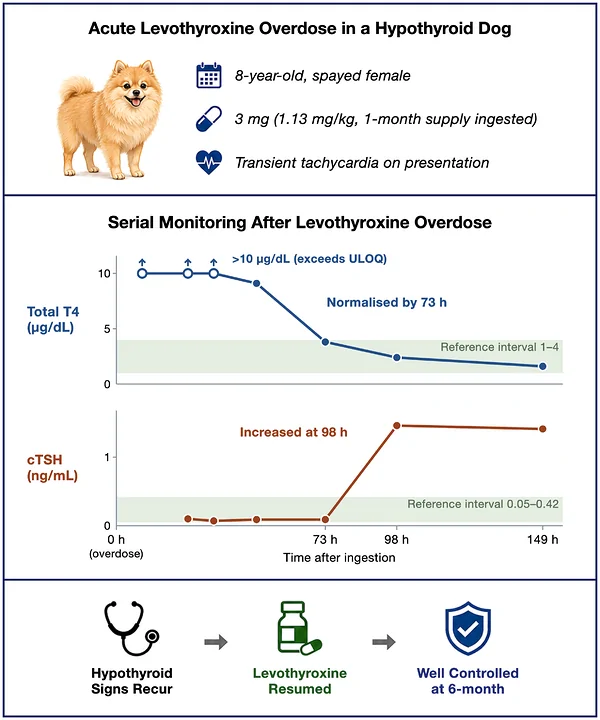

## 1 Introduction

Hypothyroidism, a common endocrine disorder in dogs, generally results from thyroid gland destruction, leading to insufficient thyroid hormone production (Chaker et al. 2022; Mooney 2023). Affected dogs present with nonspecific clinical signs, including lethargy, weakness and dermatological abnormalities (Bugbee et al. 2023). The diagnosis of hypothyroidism relies on decreased total thyroxine (T4) and increased canine thyroid‐stimulating hormone (cTSH) levels (Mooney 2023). Levothyroxine, a synthetic form of T4, remains the standard therapy for canine hypothyroidism (starting dose of 0.01–0.02 mg/kg once or twice daily) (Budde and McCluskey 2023; Nelson and Della MaggioreCouto 2019).

Thyrotoxicosis occurs when excessive circulating thyroid hormones accelerate multiple metabolic processes, causing weight loss, polyuria, polydipsia, polyphagia, tachycardia and tachypnoea (Nelson and Della MaggioreCouto 2019). In dogs, thyrotoxicosis secondary to functional thyroid tumours (Scharf et al. 2020), ingestion of meat or dietary products contaminated with thyroid tissue (Broome et al. 2015) or excessive or accidental levothyroxine administration (Fine et al. 2010; Hansen et al. 1992; Shadwick et al. 2013) has been reported.

This report describes the clinical course and outcome of acute levothyroxine overdose, along with subsequent findings during serial monitoring of total T4 and cTSH concentrations in a hypothyroid dog.

## 2 Case History

An 8‐year‐old spayed female Pomeranian (weighing 2.66 kg) was presented 9 h after accidental ingestion of an excessive dose of levothyroxine. The dog had been diagnosed with hypothyroidism 3 years earlier and was receiving oral levothyroxine (0.011 mg/kg once daily). Forty‐five days before presentation, a thyroid function test had shown decreased serum total T4 (< 0.5 µg/dL; reference interval [RI], 1–4 µg/dL; Vcheck V200, Bionote, Hwaseong‐si, Korea) and free T4 (<0.3 ng/dL; RI, 0.6–3.7 ng/dL; Greenvet Diagnostic Laboratory, Yongin‐si, Korea) concentrations, with elevated cTSH levels (1.99 ng/mL; RI, 0.05–0.42 ng/mL; Greenvet Diagnostic Laboratory). Consequently, the levothyroxine dose was increased to 50 µg (0.019 mg/kg, Synthyroxine; Dalim BioTec, Seoul, Korea) twice daily. At a recheck 18 days later, this regimen was maintained as the total T4 concentration increased to 4.34 µg/dL. However, the dog accidentally ingested a 1‐month supply of levothyroxine (total 3 mg; 1.13 mg/kg) encased in approximately 60 pill pockets (Greenies, The Nutro Company, TN, USA); the time of ingestion was designated as 0 h.

On physical examination, the dog appeared bright, alert and responsive. The respiratory rate, body temperature and systolic blood pressure were within the normal ranges, although tachycardia (180 beats/min) was noted. The results of the complete blood count and serum biochemistry were unremarkable, except for a mild increase in the aspartate aminotransferase level (64 U/L; RI, 0–50 U/L) and a triglyceride concentration exceeding the upper limit of quantification (ULOQ, > 375 mg/dL; RI, 10–100 mg/dL). Electrolyte analysis showed hypokalaemia (3.48 mmol/L; RI, 3.9–5.3 mmol/L), whereas sodium concentrations were within the RI (142 mmol/L; RI, 140–154 mmol/L). The total T4 concentration exceeded the ULOQ (> 10 µg/dL; Catalyst Dx, IDEXX, ME, USA). The dog was hospitalised for evaluation of possible thyrotoxicosis secondary to levothyroxine overdose.

To prevent further gastrointestinal absorption of the ingested levothyroxine and interrupt its enterohepatic circulation, activated charcoal (Kremezin, Kureha Chemical Industry Co., Tokyo, Japan) was administered orally at a dose of 250 mg twice daily. Concurrently, 0.9% normal saline supplemented with potassium was intravenously infused at a rate of 2.5 mL/kg/h for potassium correction. The dog maintained a good appetite and activity level with no further clinical signs of thyrotoxicosis. The initial tachycardia decreased to 127 beats/min within 1.5 h of rest, while other vital signs remained normal. Abnormalities in serum biochemistry and electrolytes were resolved by 25 h after the overdose.

The total T4 concentrations declined into the RI by 73 h, whereas cTSH concentrations (IDEXX Laboratories Inc., Seoul, Korea) remained near the lower reference limit until that time and subsequently exceeded the upper reference limit at 98 h (Table 1). At the owner's request for close monitoring, the dog remained hospitalised until Day 5 and was discharged without an immediate levothyroxine prescription because the total T4 concentration at 98 h was within the RI (2.4 µg/dL). At 2 days post‐discharge, the dog exhibited a recurrence of decreased appetite and activity, closely mirroring its initial hypothyroid presentation. Based on the clinical findings, decreased total T4 levels compared with those at discharge and persistently elevated cTSH levels (Table 1), recurrence of hypothyroidism was strongly suspected, and levothyroxine therapy was resumed. After 50 days, the total T4 (2.3 µg/dL; RI, 1–4 µg/dL; Vcheck V200), free T4 (0.9 ng/dL; RI, 0.6–3.7 ng/dL) and cTSH (0.06 ng/mL; RI, 0.05–0.42 ng/mL) concentrations (analysed by Greenvet Diagnostic Laboratory) were within the RI. The patient remained clinically stable with well‐controlled hypothyroidism during the 6‐month follow‐up.

**TABLE 1:** Serum thyroxine and thyroid‐stimulating hormone concentrations.

Variables	Time from levothyroxine overdose							
	9 h^a^	25 h	34 h	49 h	73 h	98 h	149 h^b^	RI
**Total T4** ^c^ **(µg/dL)**	> 10	> 10	> 10	9.1	3.8	2.4	1.6	1–4 µg/dL
**cTSH** ^d^ **(ng/mL)**	—	0.10	0.07	0.09	0.09	1.46	1.41	0.05–0.42 ng/mL

*Note*: — indicates not measured.

Abbreviations: cTSH, canine thyroid‐stimulating hormone; RI, reference interval; T4, thyroxine.

^a^ At presentation to our hospital.

^b^ 2 days post‐discharge.

^c^ Measured using Catalyst Dx (IDEXX, ME, USA).

^d^ Measured by IDEXX Laboratories Inc. (Seoul, Korea).

## 3 Discussion

Levothyroxine overdose has been reported in both healthy dogs and dogs with hypothyroidism (Fine et al. 2010; Hansen et al. 1992; Shadwick et al. 2013). A previous case report described two dogs that developed iatrogenic thyrotoxicosis while receiving treatment for hypothyroidism, exhibiting tachyarrhythmia, tachypnoea and agitation (Fine et al. 2010). One dog had been prescribed an excessive dose of levothyroxine for several years, and the total T4 concentration was 11.4 µg/dL (RI, 1–4 µg/dL). Another dog received double doses for 3 weeks because of caregiver miscommunication; the total T4 concentration was 75 nmol/L (RI, 15–50 nmol/L). Neither case included an assessment of the cTSH levels. In the present case, we implemented daily monitoring of total T4 and cTSH levels after a levothyroxine overdose in a hypothyroid dog.

Sequential temporal changes in serum TSH concentrations provide insights into the hypothalamic–pituitary–thyroid axis feedback during hypothyroidism, acute levothyroxine overdose and recovery. In the present case, high cTSH and low total T4 concentrations were observed during levothyroxine administration (0.011 mg/kg once daily), suggesting insufficient hypothyroidism control. The lack of profound cTSH suppression until 49 h after levothyroxine overdose might be explained by the underlying feedback mechanisms of the hypothalamic–pituitary–thyroid axis (Mendoza and Hollenberg 2017; Mooney 2023). Pituitary TSH suppression is primarily driven by intracellular T3 concentrations within the pituitary gland, which requires local deiodination of free T4–T3 rather than the circulating total T4 concentration (Mendoza and Hollenberg 2017). Consequently, this conversion process is thought to contribute to a temporal delay between the achievement of peak circulating thyroid hormone concentrations and maximal pituitary feedback. Consistent with this concept, a human patient with acute levothyroxine overdose presented with markedly elevated free T4 but normal free T3 concentrations, which resulted in TSH concentrations remaining within the lower normal limit for up to 36 h post‐ingestion (Kiran Kumar et al. 2020). Although T3 concentration was not measured in this case, these findings suggest a temporal dissociation between circulating thyroid hormone concentrations and pituitary TSH suppression. Furthermore, immediate or absolute TSH suppression is not always clinically achieved in dogs receiving levothyroxine therapy, as observed in a previous report in which two hypothyroid dogs maintained elevated cTSH concentrations (Van Dijl et al. 2014).

At presentation, the clinical findings in our case were unremarkable, despite the total T4 concentration exceeding the ULOQ (> 10 µg/dL). Transient tachycardia was considered to be associated with admission‐related stress, as it reduced within 1.5 h. Furthermore, the hypokalaemia observed at admission was likely caused by an intracellular shift in potassium. Excess circulating thyroid hormones directly stimulate Na^+^/K^+^‐ATPase pump activity on cell membranes, promoting the influx of extracellular potassium into the intracellular space (Qing et al. 2025). In humans, this mechanism is primarily associated with thyrotoxic hypokalaemic periodic paralysis; however, thyrotoxicosis‐induced hypokalaemia has not been reported in dogs. In the present case, the potassium level normalised with supplementation on the second day of hospitalisation. Clinical outcomes may differ between acute and chronic levothyroxine‐induced toxicosis. One report described a healthy dog that ingested a single 170‐mg dose of levothyroxine and vomited shortly thereafter; although total T4 concentrations were approximately 180 times the upper reference limit, no clinical signs of thyrotoxicosis were observed (Hansen et al. 1992). Conversely, another dog that developed chronic thyrotoxicosis after ingesting the faeces of a housemate dog receiving levothyroxine exhibited weight loss, polyuria, polydipsia and tachycardia despite mildly elevated total T4 concentrations (Shadwick et al. 2013). Moreover, chronic exposure to excessive thyroid hormones resulted in overt thyrotoxicosis in two hypothyroid dogs (Fine et al. 2010) and in 10 of 14 dogs that ingested thyroid hormone‐containing food (Broome et al. 2015).

Acute levothyroxine overdose is often asymptomatic or causes mild symptoms in humans (Litovitz and White 1985; Nygaard et al. 2015). Adults are more likely to develop clinical manifestations than children, reflecting differences in exposure patterns. Paediatric cases of thyrotoxicosis typically involve unintentional single‐agent overdoses during play, whereas adults often intentionally ingest excessive exogenous thyroid hormones with other medications (Nygaard et al. 2015). The human body responds to high levothyroxine doses by generating biologically inactive reverse triiodothyronine (rT3), which may mitigate toxicity through competitive receptor binding. In addition, levothyroxine binds strongly to certain plasma proteins, potentially limiting the development of thyrotoxicosis (Matthews 1993). Although increased rT3 secretion in canine thyroid tissues has been observed following exogenous T4 administration (Laurberg 1986), no in vivo study has demonstrated a compensatory inactivation response in dogs.

The oral bioavailability of levothyroxine is approximately 10%–20% in dogs and 80% in humans (Budde and McCluskey 2023; Wenzel and Kirschsieper 1977). The serum half‐life in dogs is correspondingly shorter, averaging 10–14 h compared with approximately 6.2 and 7.5 days in humans with euthyroidism and hypothyroidism, respectively (Budde and McCluskey 2023; Colucci et al. 2013). In humans, the absorption of levothyroxine tablets is reduced when they are taken with food (Wenzel and Kirschsieper 1977). Similarly, in dogs, the administration of an oral levothyroxine solution with food increased the time to achieve maximum concentrations, reduced the maximum concentration and area under the curve, and decreased the bioavailability of levothyroxine by approximately 55 ± 29% (Le Traon et al. 2008). Although our patient was administered levothyroxine tablets rather than an oral solution, co‐administration with pill pockets may have decreased their bioavailability.

The limitations of this case report include the unavailability of precise total T4 concentrations before 49 h and the absence of a comprehensive thyroid panel, including free T4 and T3 concentrations. As our patient was a small dog, collecting additional serum to remeasure T4 concentrations exceeding the ULOQ was challenging. However, total T4 was monitored as a surrogate marker for thyroid status, given its correlation with free T4 (Hare et al. 2018).

To the best of our knowledge, this is the first report to document a levothyroxine overdose in a hypothyroid dog by daily monitoring of total T4 and cTSH concentrations. The dog remained asymptomatic, and the serum total T4 concentration returned to the RI within several days of supportive therapy. These findings suggest that acute levothyroxine overdose is less severe than chronic exposure. Nevertheless, careful monitoring and appropriate preventive measures are required.

## Author Contributions

**Soomin Park**: data curation, formal analysis, writing – original draft. **Jiyeon Seo**: methodology, validation, writing – original draft. **Taeho Lee**: conceptualisation, data curation, resources, writing – review and editing. **Woonchan Ahn**: resources, writing – review and editing. **Woojin Song**: investigation, writing – review and editing. **Aryung Nam**: conceptualisation, project administration, supervision, writing – review and editing.

## Funding

The authors have nothing to report.

## Ethics Statement

Informed written consent was obtained from the owner for the publication of this case report and any accompanying clinical data.

## Conflicts of Interest

The authors declare no conflicts of interest.

## Data Availability

The data that support the findings of this study are available from the corresponding author upon reasonable request.
